# *In vitro* evolution provides insights into mechanisms of *Mycoplasma genitalium* resistance to moxifloxacin

**DOI:** 10.1093/jac/dkaf324

**Published:** 2025-09-16

**Authors:** Teck-Phui Chua, Jose L Huaman, Jennifer Danielewski, Catriona S Bradshaw, Dorothy A Machalek, Michael J McDonald, Suzanne M Garland, Gerald L Murray

**Affiliations:** Department of Obstetrics, Gynaecology and Newborn Health, University of Melbourne, Parkville, Victoria, Australia; Centre for Women’s Infectious Diseases, The Royal Women’s Hospital, Parkville, Victoria, Australia; Molecular Microbiology Research Group, Murdoch Children’s Research Institute, Parkville, Victoria, Australia; Department of Obstetrics, Gynaecology and Newborn Health, University of Melbourne, Parkville, Victoria, Australia; Centre for Women’s Infectious Diseases, The Royal Women’s Hospital, Parkville, Victoria, Australia; Molecular Microbiology Research Group, Murdoch Children’s Research Institute, Parkville, Victoria, Australia; Centre for Women’s Infectious Diseases, The Royal Women’s Hospital, Parkville, Victoria, Australia; Molecular Microbiology Research Group, Murdoch Children’s Research Institute, Parkville, Victoria, Australia; Melbourne Sexual Health Centre, Alfred Health, Carlton, Victoria, Australia; School of Translational Medicine, Monash University, Melbourne, Victoria, Australia; Centre for Epidemiology and Biostatistics, Melbourne School of Population and Global Health, University of Melbourne, Parkville, Victoria, Australia; The Kirby Institute, University of New South Wales, Sydney, New South Wales, Australia; School of Biological Sciences, Monash University, Clayton, Australia; Department of Obstetrics, Gynaecology and Newborn Health, University of Melbourne, Parkville, Victoria, Australia; Centre for Women’s Infectious Diseases, The Royal Women’s Hospital, Parkville, Victoria, Australia; Molecular Microbiology Research Group, Murdoch Children’s Research Institute, Parkville, Victoria, Australia; Department of Obstetrics, Gynaecology and Newborn Health, University of Melbourne, Parkville, Victoria, Australia; Centre for Women’s Infectious Diseases, The Royal Women’s Hospital, Parkville, Victoria, Australia; Molecular Microbiology Research Group, Murdoch Children’s Research Institute, Parkville, Victoria, Australia

## Abstract

**Background:**

Current knowledge of *Mycoplasma genitalium* fluoroquinolone resistance is based on sequence analyses of clinical samples, an approach with limited scope. Many potential resistance mutations have been described but their impact on moxifloxacin efficacy is unclear.

**Objective:**

To investigate the impact of individual mutations on fluoroquinolone resistance through selection of moxifloxacin-resistant mutants *in vitro*.

**Methods:**

*M. genitalium* G37 was passaged sequentially in sub-inhibitory concentrations of moxifloxacin. MIC values were determined for moxifloxacin, sitafloxacin, levofloxacin and ciprofloxacin. Bacterial populations were profiled using amplicon sequencing.

**Results:**

Across three independent experiments, four moxifloxacin mutants were isolated, with mutations encoding the following protein sequence variations: (i) GyrA D99Y/ParE E468K, (ii) ParC S83I/GyrA D99Y/ParE E468K, (iii) ParC D87V/GyrB P462S and (iv) GyrA M95I/ParE E468*dup*. Moxifloxacin MICs were elevated 16- to 32-fold for mutants with a single GyrA or ParC variation, and 128-fold for the dual GyrA/ParC mutant. Sitafloxacin MICs were elevated but remained lower than moxifloxacin MICs. Mutations did not have a substantial impact on *in vitro* growth dynamics. Population analysis showed that multiple mutations attained detectable population-wide frequencies, with evidence of clonal interference dynamics, with a minority becoming fixed in the population.

**Conclusion:**

Mutations in multiple genes conferring fluoroquinolone resistance appeared with regularity *in vitro*. Findings of an additive effect for ParC/GyrA changes, and greater effectiveness of sitafloxacin against resistant bacteria compared with moxifloxacin, are both consistent with clinical data. Improved understanding of fluoroquinolone resistance will inform the development of diagnostic assays predicting fluoroquinolone susceptibility.

## Introduction

*Mycoplasma genitalium* is a sexually transmitted bacterium that causes non-gonococcal urethritis in men, and cervicitis and pelvic inflammatory disease in women.^1–3^ A limited number of antibiotics are available for effective treatment. The macrolide azithromycin and the fluoroquinolone moxifloxacin are the predominant antibiotics in current use.^4^ The mechanism of resistance to macrolides is well described, conferred by point mutations in domain V of the 23S rRNA.^5^ This has enabled the implementation of diagnostic assays into clinical practice to inform treatment and improve treatment outcomes.^6,7^ Conversely, the extent to which specific mutations contribute to fluoroquinolone resistance in *M. genitalium* is not fully understood.

Fluoroquinolones target the topoisomerase IV and DNA gyrase enzymes. Both these enzymes are hetero-tetramers comprising two copies of the subunits ParC and ParE, and GyrA and GyrB, respectively.^8^ In *M. genitalium*, ParC appears to be the main target of fluoroquinolones with several amino acid changes at serine position 83 (S83) and aspartic acid position 87 (D87) identified among patients who failed fluoroquinolone treatment.^9–15^ Of these, ParC S83I is most frequently detected and has the strongest statistical association with clinical failure, with ∼60% of cases expected to fail moxifloxacin treatment.^16–18^. MIC data suggest that the moxifloxacin MIC of the S83I variant may vary from 1 to 8 mg/L.^19^ Several other changes in ParC, such as S83R/N and D87N/Y/H, have been identified and found to be less common with unknown or limited impact on MIC and treatment outcomes, warranting further investigation.^16^

In contrast to ParC, variations in GyrA are not as common and often occur in conjunction with a ParC mutation. GyrA mutations typically impact positions M95 and D99 (equivalent to ParC S83 and D87). Combining a GyrA change with ParC S83I doubles the risk of moxifloxacin failure.^14,15^ Again, multiple changes in GyrA have been described but the impact of each on fluoroquinolone MIC and treatment outcomes are not clear. Evidence for the contributions of the ParE and GyrB subunits to fluoroquinolone resistance in *M. genitalium* is scarce as few studies have analysed these two genes, and no association between clinical outcomes and *in vitro* data are available.^20–24^

While clinical studies provide valuable insights into antibiotic resistance in *M. genitalium*, they only offer a snapshot of the mutations present and emerging in clinical strains. Bacteria evolve under complex selective pressures,^25^ making it difficult to disentangle which mutations are directly responsible for resistance, and which are linked to environmental factors or genetic background. Current approaches focusing on specific target genes overlook other genomic loci that may play important roles in resistance. To address these limitations, this study took an *in vitro* approach to studying the complexity of resistance evolution; the laboratory-adapted strain *M. genitalium* G37 was sequentially passaged in sub-inhibitory concentrations of moxifloxacin to select resistant mutants for further examination.

## Materials and methods

### Bacterial strains and culture methods

The laboratory strain of *M. genitalium*, G37 (ATCC 33530), was used for all experiments. Bacteria were grown in Hayflick medium (Media Preparation Unit, Peter Doherty Institute, Melbourne, Australia), as described previously.^26^

### Antibiotics used in this study

Moxifloxacin hydrochloride (Merck, New Jersey, USA) and levofloxacin (Abcam, Cambridge, UK) were resuspended at 32 mg/mL in dimethyl sulfoxide. Sitafloxacin sesquihydrate (Merck, New Jersey, USA) was resuspended at 2 mg/mL in dimethyl sulfoxide. Ciprofloxacin (Thermo Fisher Scientific, Massachusetts, USA) was resuspended at 32 mg/mL in sterile water.

### Selection of moxifloxacin resistance mutations in vitro

Selection of antibiotic-resistant mutants was performed as previously described.^27^ Briefly, *M. genitalium* was passaged by performing sequential MIC assays with 10^3^ copies/µL of *M. genitalium* strain G37 and a 2-fold dilution of moxifloxacin (initially from 2 to 0.0625 mg/L, with an increasing concentration when the upper threshold was reached) in 1.5-mL screw cap micro tubes (Sarstedt, Nümbrecht, Germany). The MIC was determined at a threshold of 90% growth inhibition using *mgpB* qPCR,^28^ and the culture from the tube immediately below the MIC was used to inoculate the subsequent MIC assay. This was repeated until the MIC increased.

### MIC assays

MIC testing of other fluoroquinolones on the selected mutants was performed for each of the mutants in 96-well Nunclon^™^ Delta Surface plates (Thermo Fisher Scientific, Massachusetts, USA), and the MIC measured as described above.

### Detection and analysis of mutant M. genitalium

Bidirectional Sanger sequencing (Australian Genome Research Facility, Melbourne, Australia) of the *parC* and *gyrA* genes was performed periodically when the MIC increased by 4-fold or more.^14^ When a mutant was identified, isolation was performed, as described previously,^27^ for MIC testing with other fluoroquinolones and to compare growth dynamics between mutants. Dual-direction Sanger sequencing or amplicon sequencing (see next) were used to confirm the genotypes of the *parC*, *parE*, *gyrA*, and *gyrB* genes. Whole genome sequencing was also performed for each of the isolated mutants, as described before.^27^

### Population analysis of cultures during mutant selection

To examine how the *parC*, *parE*, *gyrA* and *gyrB* genes changed over time during mutant selection, each culture used to inoculate the subsequent MIC was analysed; this analysis was performed for every passage for each experiment. Each of the four genes were amplified by PCR, using previously described primers with Oxford Nanopore tails at the 5′ end of the primer, and sequenced on the Oxford Nanopore Technologies’ MinION platform (Oxford, UK), as described previously.^29^ Raw reads were basecalled using the high accuracy protocol, adapter trimmed and demultiplexed with Dorado version 0.7.2 (https://github.com/nanoporetech/dorado) before filtering by length (350 to 600 bp) and quality (quality score of at least 9) with nanoq version 0.10.0 (https://github.com/esteinig/nanoq). Filtered reads were mapped to G37 reference sequence (GenBank accession number NC_000908), using Minimap version 2.24 before variant calling on Geneious Prime version 2025.1.1 (Biomatters Ltd, Auckland, New Zealand) to determine the proportion of mutations in each gene at each passage.^27^

## Results

### Selection of moxifloxacin resistance mutations in vitro

The *M. genitalium* laboratory strain G37 was passaged in sub-inhibitory concentrations of moxifloxacin to select for resistant mutants. Three independent replicates were performed and an increase in MIC was observed in each replicate. Four mutants with different combinations of *parC*, *parE*, *gyrA* and *gyrB* mutations were isolated at various timepoints as indicated in Table 1. Whole genome sequencing was performed for these four mutants; several single nucleotide polymorphisms, deletions, insertions and indels were identified but occurred mostly in known repeat or variable regions such as adhesin genes (Tables S1–S4, available as Supplementary data at *JAC* Online).

**Table 1.** Summary of isolated moxifloxacin mutants and corresponding MIC values for several fluoroquinolones

Experiment	Passage^a^	Amino acid change (nucleotide change)				MIC (mg/L)			
		ParC	ParE	GyrA	GyrB	MOX	SFX	LEV	CIP
NA	NA	WT	WT	WT	WT	0.25	0.125	4	16
1	9	WT	E468K (G1402A)	D99Y (G295T)	WT	4	2	16	128
1	15	S83I (G248T)	E468K (G1402A)	D99Y (G295T)	WT	32	2	128	>128
2	9	D87V (A260T)	WT	WT	P462S (C1384T)	4	1	32	>128
3	4	WT	E468*dup* (1402_1404*dup*GAG)	M95I (G285A)	WT	8	1	64	>128

Additional mutations identified by whole genome sequencing are detailed in Tables S1–S4.

Nt, nucleotide; AA, amino acid; NA, not applicable; MOX, moxifloxacin; SFX, sitafloxacin; LEV, levofloxacin; CIP, ciprofloxacin; dup, duplication.

^a^Passage number when isolate was obtained.

### Relationship between amino acid changes and fluoroquinolone MICs

The MICs of antibiotics for each mutant were determined (Table 1). The dual ParC S83I/GyrA D99Y mutant had the highest MIC for moxifloxacin (128-fold higher than wild type), noting that this isolate also had a ParE E468K change. The next highest MIC was observed for the strain with a GyrA M95I change (32-fold higher than wild type), noting a ParE E468*dup* was also present. The GyrA D99Y/ParE E468K and ParC D87V/GyrB P462S mutants had the lowest MIC values (16-fold higher than wild type). In contrast, sitafloxacin MICs were 8- to 16-fold higher for all four mutants compared with wild type. As previously observed for *M. genitalium*, MICs for moxifloxacin and sitafloxacin were lower than those for levofloxacin and ciprofloxacin.

### Impact of amino acid changes on bacterial growth

**Figure 1.**
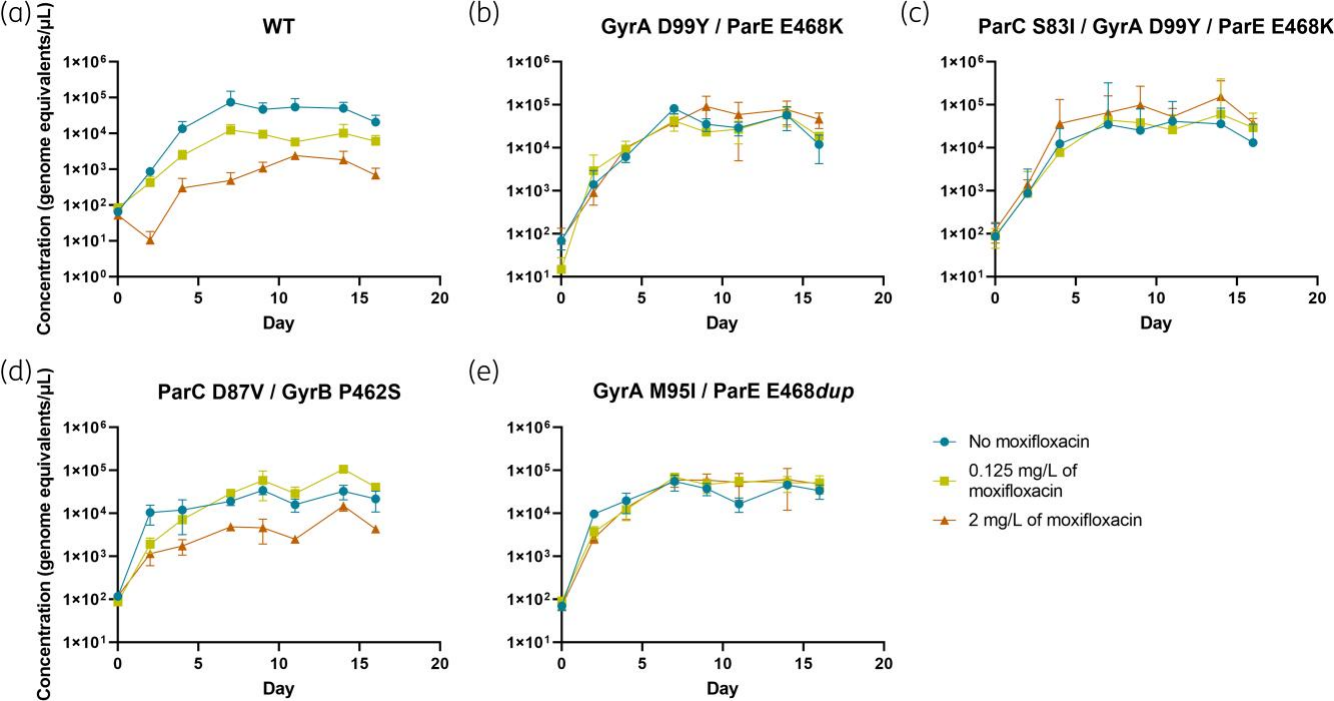
Growth dynamics of the wild type *Mycoplasma genitalium* G37 (a) and isolated mutant strains GyrA D99Y/ParE E468K (b), ParC S83I/GyrA D99Y/ParE E468K (c), ParC D87V/GyrB P462S (d) and GyrA M95I/ParE E468*dup* (e) in various concentrations of moxifloxacin (0 mg/L, 0.125 mg/L and 2 mg/L). Bacterial density was determined by quantitative PCR.

Each of the four mutant strains showed similar growth to wild type in the absence of antibiotics (Figure 1). In media containing 0.125 mg/L of moxifloxacin, the wild type G37 was moderately affected in growth but not the mutant strains. In 2 mg/L of moxifloxacin, the growth of the wild-type strain and the ParC D87V mutant were affected; there was no effect on the other three mutants.

### Population analysis of cultures during mutant selection

The evolutionary trajectories of mutations over time while under selective pressure with moxifloxacin was also investigated, with a focus on the four genes of interest: *parC*, *parE*, *gyrA* and *gyrB*.

**Figure 2.**
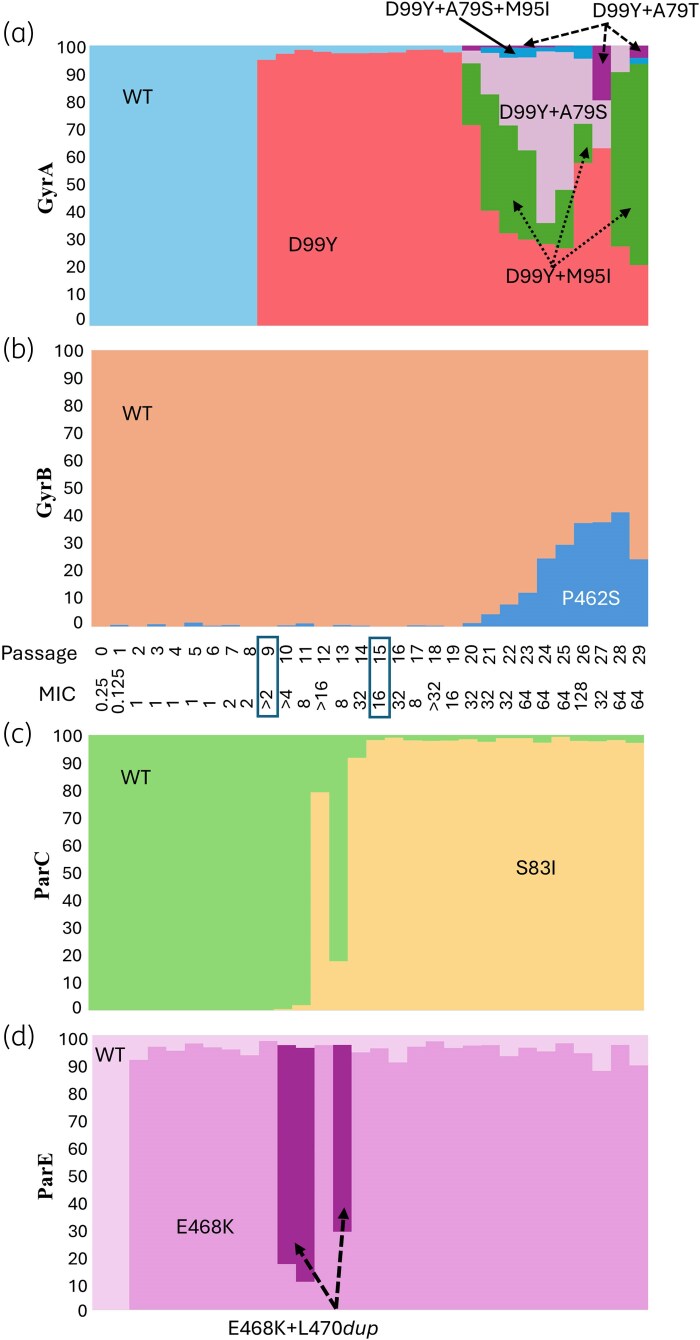
Genotypic analysis of culture populations in Experiment 1. The MIC (mg/L) of the population is indicated for each passage. The proportion of mutations in the population was determined by PCR followed by amplicon sequencing for the genes *gyrA* (a), *gyrB* (b), *parC* (c) and *parE* (d). Boxes indicate the passage where mutants were isolated corresponding to GyrA D99Y/ParE E468K and ParC S83I/GyrA D99Y/ParE E468K.

In the first experiment (Figure 2), a ParE E468K mutation appeared at passage 2 (87% of the culture; Figure 2d, mid pink). At passage 9, a GyrA D99Y mutation appeared (95%; Figure 2a, red) that coincided with an increase in MIC to >2 mg/L. At passage 20, a subpopulation of the D99Y mutants acquired an additional mutation in *gyrA*; these fluctuated in frequency until passage 28 where the D99Y + M95I mutant became the dominant genotype (Figure 2a, green). At passage 10, a large proportion of the ParE E468K mutants acquired an L470*dup* (Figure 2d, dark purple), which disappeared suddenly. Conversely, a ParC S83I mutation (Figure 2b, yellow) first appeared as the dominant mutation when the ParE E468K + L47*0dup* disappeared at passage 12 (78%) and after passage 14 (>90%); this corresponded with an increase in MIC to ≥16 mg/L.

**Figure 3.**
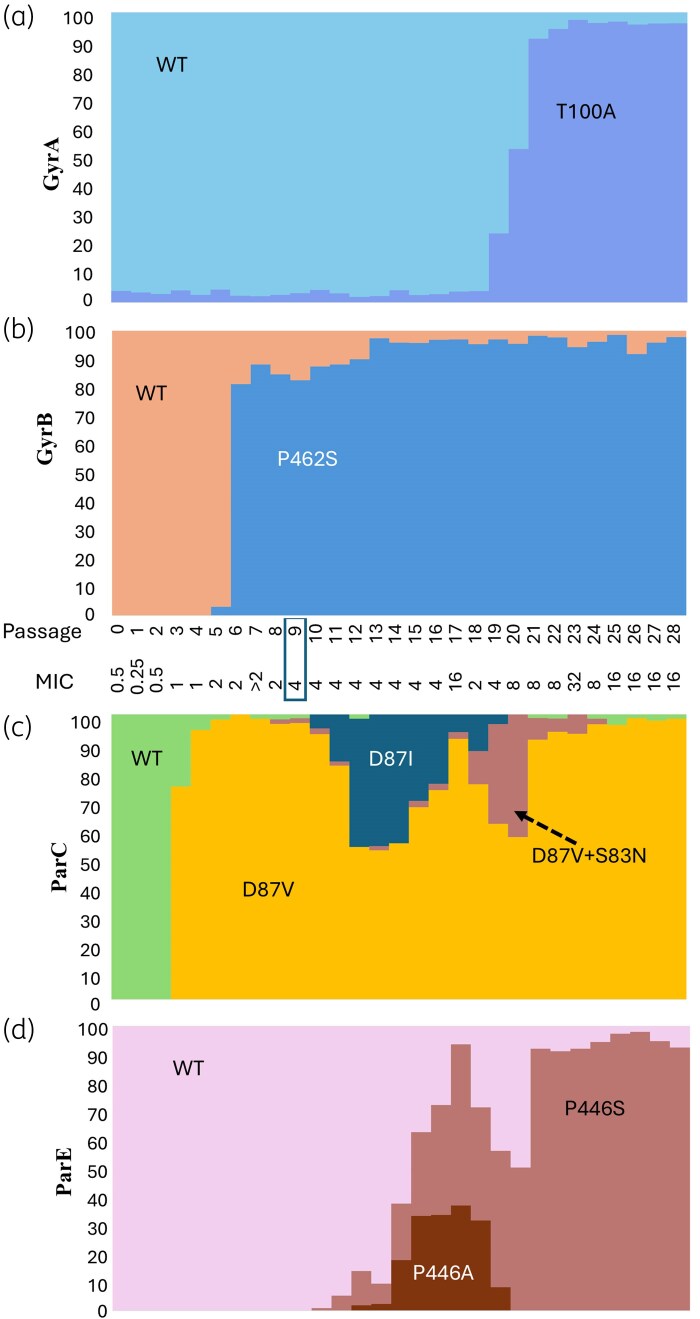
Genotypic analysis of culture populations in Experiment 2. The MIC (mg/L) of the population is indicated for each passage. The proportion of mutations in the population was determined by PCR followed by amplicon sequencing for the genes *gyrA* (a), *gyrB* (b), *parC* (c) and *parE* (d). Boxes indicate the passage where mutants were isolated corresponding to ParC D87V/GyrB P462S.

In the second experiment (Figure 3), a ParC D87V mutant first appeared at passage 3 (75%; Figure 3c, yellow), becoming the dominant variant, with a corresponding increase in MIC to 4 mg/L. A subpopulation of the D87V acquired a second mutation (D87V + S83N; Figure 3c, brown) that fluctuated, constituting up to 40% of the population, before disappearing at passage 25. At passage 10, another subpopulation of D87V mutants acquired a second mutation (G259A) resulting in a ParC D87I change (GA259–260AT; Figure 3c, dark blue); this subpopulation also increased to ∼40% before disappearing. Similarly, a ParE P446A mutation appeared at passage 12 (Figure 3d, dark brown) and increased (37%) before declining and disappearing at passage 19. By contrast, a ParE P446S mutation appeared at passage 11 (Figure 3d, mid brown) and fluctuated in frequency before stabilizing from passage 21 onwards. GyrA T100A was present in a minority (<5%) from the start of the culture and only increased at passage 19 onwards where it remained stable (96%; Figure 3a, mid blue). Interestingly, this mutation fixed at the same time as ParC D87V and ParE P446S, corresponding to a population with higher MIC than the reisolated sole D87V mutation. GyrB P462S arose at passage 5 (3%; Figure 3b, blue) then became the dominant mutation in the next passage (81%), slowly increasing to 95% and fixing.

**Figure 4.**
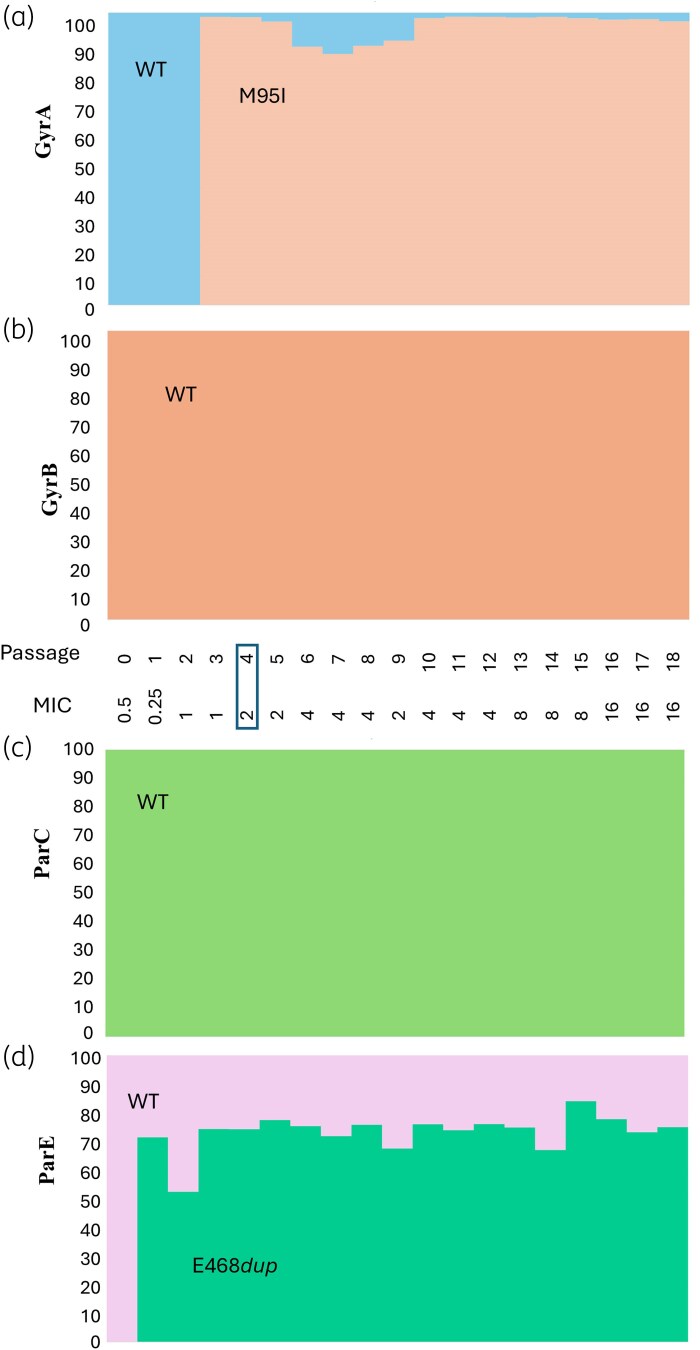
Genotypic analysis of culture populations in Experiment 3. The MIC (mg/L) of the population is indicated for each passage. The proportion of mutations in the population was determined by PCR followed by amplicon sequencing for the genes *gyrA* (a), *gyrB* (b), *parC* (c) and *parE* (d). Boxes indicate the passage where mutants were isolated, corresponding isolated strain GyrA M95I/ParE E468*dup*.

In the third experiment (Figure 4), a ParE E468*dup* appeared at the first passage (Figure 4d, green), constituting 70% of the culture, then fluctuating between 52% and 82% in subsequent passages. In the third passage, a GyrA M95I (98%; Figure 4a, tan) appeared and became fixed. The MIC of the culture increased on the appearance of the GyrA M95I mutation, stabilizing at 4–16 mg/L. Changes were not present in ParC nor GyrB.

### The appearance of linked mutations

A close analysis of the populations indicated that some mutations in different genes seem to be linked, which may indicate coincidence (without any corresponding advantage), additive impact or a form of dependence. For example, the ParC D87V change (Figure 3c, yellow) became fixed in the population along with the ParE P446S (Figure 3d, mid brown), while the ParC S83I change (Figure 2c, yellow) is coincident with the E468K change in ParE (Figure 2d, mid pink).

## Discussion

A deeper understanding of the mechanisms of fluoroquinolone resistance in *M. genitalium* is needed to inform the development of resistance assays to improve antibiotic use and cure. This study selected moxifloxacin-resistant *M. genitalium* mutants in three independent experiments. Starting with the same strain, each experiment developed a different suite of mutations, indicating a random process that was not an underlying property of the starting population. Mutants had elevated fluoroquinolone MICs 4- to 128-fold higher than wild type, with the largest impact from the dual GyrA D99Y/ParC S83I mutant. Only one mutant (ParC D87V/GyrB P462S) showed a growth defect in moxifloxacin at a concentration of 2 mg/L. During the selection process, numerous mutations appeared in each of the four genes of interest, with some disappearing after a few passages while others were fixed in the population, becoming the dominant gene variant. A previous study used a similar approach, selecting *M. genitalium* resistant mutants in the presence of ciprofloxacin, and found three mutations across *parC* and *parE* coinciding with a modest increase in MIC (although these mutations were not in key locations, such as ParC S83, D87).^20^

Four mutants with various ParC and GyrA changes were isolated after moxifloxacin exposure. ParC D87V (A260T) has been reported twice among clinical *M. genitalium* and *Chlamydia trachomatis* samples,^30,31^ while the equivalent change has been identified in non-susceptible isolates of *Streptococcus pyogenes*^32^ and *Streptococcus agalactia* (Group B *Streptococcus*).^33^ The scarcity of ParC D87V among clinical samples of *M. genitalium* and the results from this study (lower fluoroquinolone MICs and impacted growth) suggest it likely confers low-level resistance with limited clinical significance. Our findings are not unexpected as selection of resistant mutants *in vitro* involves growth in sub-inhibitory concentrations of antibiotic, so low-level resistance mutations are likely to be selected for.^34^

Clinical data suggest that GyrA changes occur rarely in isolation, and usually in combination with a ParC change (typically ParC S83I).^14,15,35^ Therefore, it was unexpected to identify two GyrA mutants (D99Y and M95I) without a ParC change, and a ParC S83I change appearing after continuous passage of one of the GyrA mutants. However, *in vitro* models of resistance generation do not always replicate that *in vivo*, as seen in the previous study on *M. genitalium* which selected ParC G81C, ParC P26T, and ParE N266K using ciprofloxacin.^20^ Other factors during treatment likely contribute to the preferential selection of ParC mutants over GyrA mutants clinically. Of the two individual GyrA changes described in this study, the GyrA M95I mutation showed a higher impact on MIC compared with the GyrA D99Y change; however, it is important to note both mutants had different ParE changes. Although the extent to which ParE and GyrB contribute individually to fluoroquinolone resistance could not be elucidated in this study, the induction of mutations in these two genes under moxifloxacin selection pressure suggests they have a role in resistance, either directly or indirectly (e.g. secondary suppressor mutation).

The dual ParC/GyrA mutant had the highest level of resistance. Whether this was due to the presence of ParC S83I or the presence of both a ParC and GyrA change could not be determined as a sole ParC S83I variant was not available for comparison. Nevertheless, the sequential acquisition of mutations and stepwise elevation of MIC provides compelling evidence of an additive effect; this is consistent with clinical studies showing that if a ParC S83I change is present with a GyrA change, the risk of moxifloxacin and sitafloxacin failure is doubled^14,15,35^ and the MIC is higher (nearly 2-fold higher for moxifloxacin and 2.5-fold higher for sitafloxacin)^19,36^ than those with a ParC S83I change alone. The impact of a sole ParC S83I change remains of interest because it is the most common mutation among clinical samples that has increased in frequency over time,^17^ it is the strongest predictor of fluoroquinolone failure^14–16,18^ and, from limited culture data, it has the biggest individual impact on MIC compared with other ParC changes.^19^

The small number of replicates in this study limits the ability to analyse population changes under antibiotic selective pressure, but some speculation is possible. Mutations in the *parC* or *parE* genes were the first to become dominant in the population. Where *gyrA* and *gyrB* mutations arose early (first and second experiments), they remained at low frequencies initially, suggesting a fitness cost, but this cost may have been alleviated by additional mutations in other genes later in the experiment. In populations with multiple *parC* or *parE* mutations, each variant arose independently on different genetic backgrounds, resulting in competition through clonal interference rather than cumulative mutations within a single lineage.

The dynamic changes in mutations (appearance, disappearance, fixing in the population) has been seen in other evolution experiments.^34,37^ The degree of variation may be attributed to a lack of DNA repair proteins in *M. genitalium* (conferring a high mutation rate),^38^ in addition to the mode of fluoroquinolone action (impaired DNA replication).^8^ Fluctuations of mutant alleles seen throughout the experiments could be accounted by a balance between fitness cost of the mutation and selective advantage, where a mutant is unable to grow rapidly unless additional selection pressure is applied, or reversion of some mutants into wild type as proposed by two clinical studies.^15,35^ In contrast, wild-type alleles persisted across all populations, never declining to zero frequency. One plausible explanation is collective antibiotic resistance, where resistant cells reduce the effective antibiotic concentration in the environment, thereby shielding susceptible wild type cells.^39^ This communal protection can create conditions conducive to frequency-dependent selection, wherein the fitness advantage of resistance diminishes as its prevalence increases, which allows wild type cells to persist.^40^ While spatial refugia (i.e. microenvironments with lower antibiotic concentrations) can also facilitate wild-type survival^41^ this mechanism is less likely in our experiments due to the homogeneous mixing conditions employed.

There are limitations to this study. The laboratory strain of G37 was not reisolated before use. However, this reflects the genetic diversity probably present in a natural infection. The common co-occurrence of mutations meant resolving the impact of individual mutations was not always possible. This study focused on moxifloxacin because it is the most important fluoroquinolone treatment option, but development of resistance mutations may differ depending on the fluoroquinolone (e.g. ciprofloxacin^20^). The induction of additional mutants will be very useful in gaining a thorough understanding of the mutations leading to clinical failure of fluoroquinolones.

In conclusion, this study demonstrated the feasibility of *in vitro* selection of moxifloxacin-resistant *M. genitalium* to study the mechanisms of resistance development. Findings support clinical data showing a greatly increased risk of fluoroquinolone failure if a dual ParC/GyrA change is present. Analysis of ParE and GyrB indicate that mutations in these genes are unlikely coincidental, thus their impact on resistance should not be overlooked. Population analysis found mixtures of wild type and mutant alleles present throughout continuous exposure to sub-inhibitory concentrations of moxifloxacin, providing a snapshot of the development of fluoroquinolone resistance over time and revealing potential fitness costs. Further investigation of the sequence in which resistance mutations arise enables better design of treatment strategies to slow or prevent resistance development and guide proactive antibiotic usage policies. With limited alternative therapies available, predicting evolutionary trajectories is key to ensuring the long-term efficacy of current treatments.

## Supplementary Material

dkaf324_Supplementary_Data

## Supplementary data

Tables S1 to S4 are available as Supplementary data at *JAC* Online.
