# Supplementary material for: In vitro evolution provides insights into mechanisms of Mycoplasma genitalium resistance to moxifloxacin
Source: J Antimicrob Chemother. 2025 Sep 16;80(11):2970–7. doi: 10.1093/jac/dkaf324 (PMC12596053; doi:10.1093/jac/dkaf324)
Supplement: dkaf324_Supplementary_Data [file dkaf324_supplementary_data.docx]

**Supplementary Table 1. Summary of nucleotide changes in the GyrA D99Y mutant (Experiment 1; whole genome analysis)**

| **Gene/Protein (locus tag)** | **Function** | **G37 genome location*** | **Nucleotide change** | **Amino acid change** | **Comments** |
| --- | --- | --- | --- | --- | --- |
| **Coding regions** | | | | | |
| *gyrA*  (MG_RS00020) | Negative supercoiling of circular dsDNA | 5,106 | G to T | D99Y |  |
| DUF240 domain-containing protein  (MG_RS00530) | Unknown | 121,851 | A del | E94fsX96 | Repeat A region; premature stop codon |
| ABC transporter permease  (MG_RS00655) | Membrane transport | 147,459 | G del | M262fsX284 | Premature stop codon |
| *parE*  (MG_RS01150) | Decatenation of chromosomes and relaxing supercoiled DNA | 241,724 | G to A | E468K |  |
| Hypothetical protein  (MG_RS02905) | Unknown | 430,015-7 | CTA del | L137del | Repeat region |
| Hypothetical protein  (MG_RS02415) | Unknown | 500,853-4 | GAC ins | S522_H523insR |  |
| *pstS*  (MG_RS02490) | Subunit of phosphate ABC transporter | 515,822 | T del | N101fsX110 | Repeat T region; premature stop codon |
| **Hypervariable regions** | | | | | |
| Adhesin (MG_RS00380) | Adhesion | 86,400 | G to A | Silent |  |
|  |  | 86,430 | A to G | Silent |  |
|  |  | 86,431 | A to C | Silent |  |
|  |  | 86,433 | A to C |  |  |
|  |  | 86,436 | C to T | Silent |  |
|  |  | 86,452-4 | GCA to AGC | A107S |  |
|  |  | 86,455 | C to T | Silent |  |
|  |  | 86,457 | G to A |  |  |
|  |  | 86,458 | T to C | Silent |  |
|  |  | 86,466 | G to T | Silent |  |
|  |  | 86,469 | C to T | Silent |  |
|  |  | 86,475 | A to C | Silent |  |
|  |  | 86,493 | A to G | Silent |  |
|  |  | 86,495-6 | GA to AT | G121D |  |
|  |  | 86,500-2 | GGG to AAT | G123N |  |
|  |  | 86,503 | A to G | N124D |  |
|  |  | 86,506 | G to C | E125Q |  |
|  |  | 86,508 | G to A |  |  |
|  |  | 86,513 | C to A | T127N |  |
|  |  | 86,523 | C to A | H128K |  |
|  |  | 86,517 | T to G |  |  |
|  |  | 86,518-20 | ACG to GAT | T129D |  |
|  |  | 86,530 | C to A | Q133K |  |
|  |  | 86,532 | G to A |  |  |
|  |  | 86,535 | A to G | Silent |  |
|  |  | 86,536 | A to G | N135D |  |
|  |  | 86,538 | C to T |  |  |
|  |  | 86,543 | TG ins | D138Efs142 |  |
|  |  | 86,547 | T to C | K139T |  |
|  |  | 86,552 | T to G | L140N |  |
|  |  | 86,555-6 | GA del | G141E |  |
|  |  | 86,562 | C to T | Silent |  |
|  |  | 86,563 | C to T | Silent |  |
|  |  | 86,565 | C to A |  |  |
|  |  | 86,568 | G to T | Silent |  |
| mgpA  (MG_RS01075) | Adhesion | 224,257 | A to G | Silent |  |
|  |  | 224,287 | G to A | Silent |  |
|  |  | 224,288 | C to A | Silent |  |
|  |  | 224,290 | C to A |  |  |
|  |  | 224,293 | T to C | Silent |  |
|  |  | 224,309-11 | AGC to GCA | S914A |  |
|  |  | 224,312 | T to C | Silent |  |
|  |  | 224,314 | A to G |  |  |
|  |  | 224,315 | C to T | Silent |  |
|  |  | 224,323 | T to G | Silent |  |
|  |  | 224,326 | T to C | Silent |  |
|  |  | 224,332 | C to A | Silent |  |
|  |  | 224,350 | G to A | Silent |  |
|  |  | 224,352-3 | AT to GA | D928G |  |
|  |  | 224,357-9 | AAT to GGG | N930G |  |
|  |  | 224,360 | G to A | D931N |  |
|  |  | 224,363 | C to G | Q932E |  |
|  |  | 224,365 | A to G |  |  |
|  |  | 224,370 | A to C | N934T |  |
|  |  | 224,372 | A to C | K935H |  |
|  |  | 224,374 | G to T |  |  |
|  |  | 224,375-7 | GAT to ACG | D936T |  |
|  |  | 224,387 | A to C | K940Q |  |
|  |  | 224,389 | A to G |  |  |
|  |  | 224,392 | G to A | Silent |  |
|  |  | 224,393 | G to A | D942N |  |
|  |  | 224,395 | T to C |  |  |
|  |  | 224,396 | G to A | K943E |  |
|  |  | 224,401-2 | TG del | E945Dfs948 |  |
|  |  | 224,406 | C to T | T946K |  |
|  |  | 224,411 | G to T | N947L |  |
|  |  | 224,413-4 | GA ins | E948G |  |
|  |  | 224,419 | T to C | Silent |  |
|  |  | 224,420 | T to C | Silent |  |
|  |  | 224,422 | A to C |  |  |
|  |  | 224,425 | T to G | Silent |  |
| P110  (MG_RS01080) | Adhesion | 227,130-2 | AGT dup | S206dup | Repeat AGT region |
| MgpC  (MG_RS01730) | Adhesion | 351,454-6 | AGT dup | S337dup | Repeat AGT region |
| MgpC  (MG_RS02070) | Adhesion | 429,304-5 | AGT dup | S334dup | Repeat AGT region |
| **Non-coding regions** | | | | | |
| Non-coding region | N/A | 36,791 | A ins | N/A | Repeat A region |
| Non-coding region | N/A | 306,212-3 | AT ins | N/A |  |

*GenBank accession NC_000908

**Supplementary Table 2. Summary of nucleotide changes in the ParC S83I/GyrA D99Y mutant (Experiment 1; whole genome analysis)**

| **Gene/Protein** | **Function** | **G37 genome location*** | **Nucleotide change** | **Amino acid change** | **Comments** |
| --- | --- | --- | --- | --- | --- |
| **Coding regions** | | | | | |
| *gyrA*  (MG_RS00020) | Negative supercoiling of circular dsDNA | 5,106 | G to T | D99Y |  |
| DUF240 domain-containing protein  (MG_RS00530) | Unknown | 121,851 | A del | E94fsX96 | Repeat A region; premature stop codon |
| ABC transporter permease  (MG_RS00655) | Membrane transport | 147,459 | G del | M262fsX284 | Premature stop codon |
| *parE*  (MG_RS01150) | Decatenation of chromosomes and relaxing supercoiled DNA | 241,724 | G to A | E468K |  |
| *parC*  (MG_RS01155) | Decatenation of chromosomes and relaxing supercoiled DNA | 242,471 | G to T | S83I |  |
| Hypothetical protein  (MG_RS02905) | Unknown | 430,012-7 | CTTCTA del | L136_L137del | Repeat region |
| *pstS*  (MG_RS02490) | Subunit of phosphate ABC transporter | 515,822 | T del | N101fsX110 | Repeat T region; premature stop codon |
| ABC transporter permease  (MG_RS02750) | Membrane transport | 573,724 | T to A | R874S |  |
| **Hypervariable regions** | | | | | |
| Adhesin  (MG_RS00380) | Adhesion | 86,400 | G to A | Silent |  |
|  |  | 86,430 | A to G | Silent |  |
|  |  | 86,431 | A to C | Silent |  |
|  |  | 86,433 | A to C |  |  |
|  |  | 86,436 | C to T | Silent |  |
|  |  | 86,452-4 | GCA to AGC | A107S |  |
|  |  | 86,455 | C to T | Silent |  |
|  |  | 86,457 | G to A |  |  |
|  |  | 86,458 | T to C | Silent |  |
|  |  | 86,466 | G to T | Silent |  |
|  |  | 86,469 | C to T | Silent |  |
|  |  | 86,475 | A to C | Silent |  |
|  |  | 86,493 | A to G | Silent |  |
|  |  | 86,495-6 | GA to AT | G121D |  |
|  |  | 86,500-2 | GGG to AAT | G123N |  |
|  |  | 86,503 | A to G | N124D |  |
|  |  | 86,506 | G to C | E125Q |  |
|  |  | 86,508 | G to A |  |  |
|  |  | 86,513 | C to A | T127N |  |
|  |  | 86,523 | C to A | H128K |  |
|  |  | 86,517 | T to G |  |  |
|  |  | 86,518-20 | ACG to GAT | T129D |  |
|  |  | 86,530 | C to A | Q133K |  |
|  |  | 86,532 | G to A |  |  |
|  |  | 86,535 | A to G | Silent |  |
|  |  | 86,536 | A to G | N135D |  |
|  |  | 86,538 | C to T |  |  |
|  |  | 86,543 | TG ins | D138Efs4 |  |
|  |  | 86,547 | T to C | K139T |  |
|  |  | 86,552 | T to G | L140N |  |
|  |  | 86,555-6 | GA del | G141E |  |
|  |  | 86,562 | C to T | Silent |  |
|  |  | 86,563 | C to T | Silent |  |
|  |  | 86,565 | C to A |  |  |
|  |  | 86,568 | G to T | Silent |  |
| MgpC  (MG_RS00770) | Adhesion | 169,089 | C to T | Silent |  |
|  |  | 169,103-4 | GG to AT | G209D |  |
|  |  | 169,105 | G to C | D210Q |  |
|  |  | 169,107 | T to A |  |  |
|  |  | 169,108-10 | GGA to ACC | G211T |  |
|  |  | 169,111-3 | ATG to CCA | M212P |  |
|  |  | 169,116 | G to A | Silent |  |
|  |  | 169,122 | G to T | Silent |  |
|  |  | 169,123 | G to A | V216I |  |
|  |  | 169,125 | T to C |  |  |
|  |  | 169,149 | C to T | Silent |  |
|  |  | 169,210 | T to A | L245M |  |
|  |  | 169,479-81 | AGT dup | S335dup | Repeat AGT region |
| *mgpA*  (MG_RS01075) | Adhesion | 224,257 | A to G | Silent |  |
|  |  | 224,287 | G to A | Silent |  |
|  |  | 224,288 | C to A | Silent |  |
|  |  | 224,290 | C to A |  |  |
|  |  | 224,293 | T to C | Silent |  |
|  |  | 224,309-11 | AGC to GCA | S914A |  |
|  |  | 224,312 | T to C | Silent |  |
|  |  | 224,314 | A to G |  |  |
|  |  | 224,315 | C to T | Silent |  |
|  |  | 224,323 | T to G | Silent |  |
|  |  | 224,326 | T to C | Silent |  |
|  |  | 224,332 | C to A | Silent |  |
|  |  | 224,350 | G to A | Silent |  |
|  |  | 224,352-3 | AT to GA | D928G |  |
|  |  | 224,357-9 | AAT to GGG | N930G |  |
|  |  | 224,360 | G to A | D931N |  |
|  |  | 224,363 | C to G | Q932E |  |
|  |  | 224,365 | A to G |  |  |
|  |  | 224,370 | A to C | N934T |  |
|  |  | 224,372 | A to C | K935H |  |
|  |  | 224,374 | G to T |  |  |
|  |  | 224,375-7 | GAT to ACG | D936T |  |
|  |  | 224,387 | A to C | K940Q |  |
|  |  | 224,389 | A to G |  |  |
|  |  | 224,392 | G to A | Silent |  |
|  |  | 224,393 | G to A | D942N |  |
|  |  | 224,395 | T to C |  |  |
|  |  | 224,396 | G to A | K943E |  |
|  |  | 224,401-2 | TG del | E945Dfs948 |  |
|  |  | 224,406 | C to T | T946K |  |
|  |  | 224,411 | G to T | N947L |  |
|  |  | 224,413-4 | GA ins | E948G |  |
|  |  | 224,419 | T to C | Silent |  |
|  |  | 224,420 | T to C | Silent |  |
|  |  | 224,422 | A to C |  |  |
|  |  | 224,425 | T to G | Silent |  |
|  |  | 224,985-6 | GT to AC | S1139N |  |
| P110  (MG_RS01080) | Adhesion | 226,742 | T to C | Silent |  |
|  |  | 226,756-7 | AT to GG | D281G |  |
|  |  | 226,758 | C to G | Q282D |  |
|  |  | 226,760 | A to T |  |  |
|  |  | 226,761-3 | ACC to GGA | T283G |  |
|  |  | 226,764-6 | CCA to ATG | M284P |  |
|  |  | 226,769 | A to G | Silent |  |
|  |  | 226,775 | T to G | Silent |  |
|  |  | 226,776 | A to G | V288I |  |
|  |  | 226,778 | C to T |  |  |
|  |  | 226,802 | T to C | Silent |  |
|  |  | 226,863 | A to T | M317L |  |
|  |  | 226,893-5 | CAG to ACA | T327Q |  |
|  |  | 226,896-8 | GAA to ACT | T328E |  |
|  |  | 226,899 | A to G | N329D |  |
|  |  | 226,902 | G to T | A330S |  |
|  |  | 226,905-7 | AGT to TCA | Silent |  |
|  |  | 226,908-10 | AAC to TCA | N332S |  |
|  |  | 226,941 | G to A | D343N |  |
|  |  | 226,949 | A to G | Silent |  |
|  |  | 227,087 | A to G | Silent |  |
|  |  | 227,088 | A to T | I392L |  |
|  |  | 227,090 | C to G |  |  |
|  |  | 227,130-2 | AGT dup | S206dup | Repeat AGT region |
|  |  | 227,227 | A to G | K438R |  |
|  |  | 227,333-4 | TCA ins | N473_N474insS |  |
|  |  | 227,339 | A to T | Silent |  |
|  |  | 227,340 | A to T | T476S |  |
|  |  | 227,343 | A to T | T477S |  |
|  |  | 227,345 | T to A |  |  |
| MgpC  (MG_RS01730) | Adhesion | 351,217-9 | ACA to CAG | T258Q |  |
|  |  | 351,220-2 | ACT to GAA | T259E |  |
|  |  | 351,223 | G to A | D260N |  |
|  |  | 351,226 | T to G | S261A |  |
|  |  | 351,229-31 | TCA to AGT | Silent |  |
|  |  | 351,232-4 | TCA to AAC | S263N |  |
|  |  | 351,265 | A to G | N274D |  |
|  |  | 351,273 | G to A | Silent |  |
|  |  | 351,279 | T to C | Silent |  |
|  |  | 351,411 | G to A | Silent |  |
|  |  | 351,412 | T to A | L323I |  |
|  |  | 351,414 | G to C |  |  |
|  |  | 351,454-6 | AGT dup | S337dup | Repeat AGT region |
|  |  | 351,545 | G to A | R367K |  |
|  |  | 351,652-4 | TCA del | S403del |  |
|  |  | 351,660 | T to A | Silent |  |
|  |  | 351,661 | T to A | S406T |  |
|  |  | 351,664 | T to A | S407T |  |
|  |  | 351,666 | A to T |  |  |
|  |  | 351,670 | A to C | T409P |  |
|  |  | 351,681 | G to T | Silent |  |
| **Non-coding regions** | | | | | |
| Non-coding region | N/A | 36,791-3 | AA del | N/A | Repeat A region |
| Non-coding region | N/A | 175,418 | T to C | N/A |  |
|  |  | 175,426-7 | AC to GT | N/A |  |
| Non-coding region | N/A | 306,212-3 | AT ins | N/A |  |

*GenBank accession NC_000908

**Supplementary Table 3. Summary of nucleotide changes in the ParC D87V mutant (Experiment 2; whole genome analysis)**

| **Gene/Protein** | **Function** | **G37 genome location*** | **Nucleotide change** | **Amino acid change** | **Comments** |
| --- | --- | --- | --- | --- | --- |
| **Coding regions** | | | | | |
| *gyrB*  (MG_RS00015) | Negative supercoiling of circular dsDNA | 4,228 | C to T | P462S |  |
| ATP-binding protein  (MG_RS00650) | Membrane transport (ABC transporter) | 146,381 | G to T | G470V |  |
| *parC*  (MG_RS01155) | Decatenation of chromosomes and relaxing supercoiled DNA | 242,483 | A to T | D87V |  |
| DUF3196 family protein  (MG_RS01390) | Unknown | 281,324 | T del | G82fsX90 | Repeat T region |
| Hypothetical protein  (MG_RS01525) | Unknown | 313,037 | A to T | Silent |  |
| Hypothetical protein  (MG_RS01595) | Unknown | 326,686-7 | C dup | Q123fsX137 | Premature stop codon |
| Protein M  (MG_RS01660) | Immunoglobulin-binding protein | 342,115 | C to A | G530N |  |
| DUF3713 domain-containing protein  (MG_RS01850) | Unknown | 384,463-8 | ACTATT del | N648_S649del | Repeat ACTATT region |
| DUF3713 domain-containing protein  (MG_RS02060) | Unknown | 425,827-9 | TGT del | T362del | Repeat TGT region |
| *pstA*  (MG_RS02485) | Subunit of phosphate ABC transporter | 514,750 | A del | L81fsX | Premature stop codon |
| **Hypervariable regions** | | | | | |
| Adhesin  (MG_RS00765) | Adhesion | 167,849 | T to C | Silent |  |
|  |  | 167,852 | T to C | Silent |  |
|  |  | 167,855 | A to C | E42D |  |
|  |  | 168,030 | T to C | Silent |  |
|  |  | 168,032 | A to C |  |  |
|  |  | 168,036-8 | GCA to AGC | S103A |  |
|  |  | 168,039 | C to T | Silent |  |
|  |  | 168,041 | G to A |  |  |
|  |  | 168,042 | T to C | Silent |  |
| MgpC  (MG_RS00770) | Adhesion | 169,482-3 | AGT dup | S335dup | Repeat AGT region |
| Adhesin  (MG_RS02810) | Adhesion | 213,598 | T to C | V8A |  |
|  |  | 213,600 | G to T | V9L |  |
|  |  | 213,602 | G to A |  |  |
|  |  | 213,654 | T to G | S27A |  |
|  |  | 213,656 | T to A |  |  |
|  |  | 213,657 | T to A | S28T |  |
|  |  | 213,660-2 | TCC to GAT | S29D |  |
|  |  | 213,668 | C to T | Silent |  |
|  |  | 213,669 | A to G | N32D |  |
|  |  | 213,671 | C to T |  |  |
|  |  | 213,681 | A to G | R36G |  |
|  |  | 213,701 | G to A | Silent |  |
|  |  | 213,715-6 | AC to GT | N47S |  |
|  |  | 213,720 | G to C | D49Q |  |
|  |  | 213,722 | C to A |  |  |
|  |  | 213,723 | A to G | S50G |  |
|  |  | 213,725 | T to G |  |  |
| *mgpA*  (MG_RS01075) | Adhesion | 222,198 | C to T | A210V |  |
|  |  | 222,200 | T to G | L211V |  |
|  |  | 222,202 | A to G |  |  |
|  |  | 222,254 | G to T | A229S |  |
|  |  | 222,256 | A to T |  |  |
|  |  | 222,257-62 | delACCTGA  insTCT | T230_W231delinsS |  |
|  |  | 222,271 | T to C | Silent |  |
|  |  | 222,272 | G to A | D235N |  |
|  |  | 222,274 | T to C |  |  |
|  |  | 222,284 | G to A | G239R |  |
|  |  | 222,304 | A to G | Silent |  |
|  |  | 222,365 | A to G | S266G |  |
|  |  | 222,371-2 | AT to GG | M268G |  |
|  |  | 222,379 | C to A | Silent |  |
|  |  | 222,383 | A to C | T272P |  |
|  |  | 222,385 | A to T |  |  |
|  |  | 222,386-91 | delAGAGAT  insCGC | R273_D274del  insR |  |
|  |  | 222,402-3 | AA to CC | K278T |  |
|  |  | 222,404 | G to A | V279I |  |
|  |  | 222,406 | G to T |  |  |
|  |  | 222,457 | T to C | Silent |  |
|  |  | 222,469 | A to G | Silent |  |
|  |  | 222,472 | G to A | Silent |  |
|  |  | 222,502 | A to G | Silent |  |
|  |  | 222,505 | G to T | E312D |  |
|  |  | 222,508 | A to G | Silent |  |
|  |  | 222,509-11 | GAG to AGT | E314S |  |
|  |  | 222,512-3 | AA to GG | K315G |  |
|  |  | 224,059 | G to A | Silent |  |
|  |  | 224,068 | G to T | Silent |  |
|  |  | 224,072-86 | delCGCGTT  CAAAGGGTT  insAGAAAA  GCT | R825_V839del  insRKA |  |
|  |  | 224,087 | C to A | H840N |  |
|  |  | 224,092-3 | AAC dup | N841dup |  |
|  |  | 224,094 | C to A | T842N |  |
|  |  | 224,095 | A to T |  |  |
| Hypothetical protein  (MG_RS01720) | Unknown | 349,456-8 | GGT del | G62del |  |
|  |  | 349,462-3 | GG to AT | G64M |  |
|  |  | 349,470 | A to C | Silent |  |
|  |  | 349,474 | C to A | P68T |  |
|  |  | 349,476 | T to A |  |  |
|  |  | 349,477 | C to A | R69_K70insD |  |
|  |  | 349,478-9 | AGA ins |  |  |
|  |  | 349,479 | C to A |  |  |
|  |  | 349,490-1 | CC to AA | T73K |  |
|  |  | 349,492 | A to G | I74V |  |
|  |  | 349,494 | T to G |  |  |
|  |  | 349,545 | C to T | Silent |  |
|  |  | 349,557 | G to A | Silent |  |
|  |  | 349,560 | A to G | Silent |  |
|  |  | 349,590 | G to A | Silent |  |
|  |  | 349,593 | T to G | D107E |  |
|  |  | 349,596 | G to A | Silent |  |
|  |  | 349,597-9 | AGT to GAG | S109E |  |
|  |  | 349,600-1 | GG to AA | G110K |  |
| **Non-coding regions** | | | | | |
| Non-coding region | N/A | 36,794-7 | AAAAAA del | N/A | Repeat A region |
| Non-coding region | N/A | 546,193 | T del | N/A | Repeat T region |

*GenBank accession NC_000908

**Supplementary Table 4. Summary of nucleotide changes in the GyrA M95I mutant (Experiment 3; whole genome analysis)**

*GenBank accession NC_000908

| **Gene/Protein** | **Function** | **G37 genome location*** | **Nucleotide change** | **Amino acid change** | **Comments** |
| --- | --- | --- | --- | --- | --- |
| **Coding regions** | | | | | |
| *gyrA*  (MG_RS00020) | Negative supercoiling of circular dsDNA | 5,096 | G to A | M95I |  |
| ATP-binding cassette  (MG_RS00210) | Membrane transport | 50,299 | G to A | E154K |  |
| Hypothetical protein  (MG_RS01095) | Unknown | 231,167 | G to T | G187W |  |
| *parE*  (MG_RS01150) | Decatenation of chromosomes and relaxing supercoiled DNA | 241,724-6 | GAG dup | E468dup |  |
| Hypothetical protein  (MG_RS01315) | Unknown | 269,183-4 | A ins | P391fsX411 | Repeat A region; premature stop codon |
| DUF3196 family protein  (MG_RS01390) | Unknown | 281,243-4 | A ins | K56fsX60 | Repeat A region; premature stop codon |
| *IpdA*  (MG_RS01610) | Subunit of dihydrolipoyl dehydrogenase | 329,636 | G to T | A64C |  |
| *alaS*  (MG_RS01765) | Alanine tRNA ligase | 359,589-90 | T ins | K335fsX333 | Repeat T region |
| Hypothetical protein  (MG_RS02905) | Unknown | 429,993-7 | CTT del | L130del | Repeat CTT region |
|  |  | 430,017 | A to T | Silent |  |
| **Hypervariable regions** | | | | | |
| MgpC  (MG_RS00770) | Adhesion | 169,482-3 | AGT dup | S335dup | Repeat AGT region |
| MgpC  (MG_RS02070) | Adhesion | 429,306-8 | AGT del | S334del | Repeat AGT region |
| **Non-coding region** | | | | | |
| Non-coding region | N/A | 36,794 | A del | N/A |  |
